# Safety of Prolonged Wait Time for Nephrectomy for Clinically Localized Renal Cell Carcinoma

**DOI:** 10.3389/fonc.2021.617383

**Published:** 2021-03-30

**Authors:** Nienie Qi, Fangzheng Zhao, Xiaoxiao Liu, Wei Wei, Junqi Wang

**Affiliations:** ^1^ Department of Urology, The Affiliated Hospital of Xuzhou Medical University. Xuzhou Medical University, Xuzhou, China; ^2^ Department of Radiation Oncology, Cancer Center, The Affiliated Hospital of Xuzhou Medical University, Xuzhou, China; ^3^ Jiangsu Center for the Collaboration and Innovation of Cancer Biotherapy, Cancer Institute, Xuzhou Medical University, Xuzhou, China; ^4^ Department of Health Management Center, The Affiliated Hospital of Xuzhou Medical University, Xuzhou, China

**Keywords:** renal cell carcinoma, surgery, wait time, nephrectomy, interval

## Abstract

**Background:**

There is usually a surgical wait time before nephrectomy for patients with clinically localized renal cell carcinoma, and many factors can influence this preoperative wait time. A relatively prolonged wait time may cause tumor progression. Therefore, we assessed the effect of preoperative wait time on the prognosis of patients with clinically localized renal cell carcinoma.

**Methods:**

The outcomes of 561 patients with clinically localized renal cell carcinoma who underwent nephrectomy between July 2011 and March 2017 were retrospectively evaluated. According to the wait time before surgery, we divided the patients into three groups: short-wait group (≤ 30 days), intermediate-wait group (> 30 and ≤ 90 days), and long-wait group (>90 days). The clinicopathological characteristics were evaluated, and the survival rates of the three groups were compared.

**Results:**

This study included 370 male (66%) and 191(34%) female patients, with a median age of 64 years. There were 520 patients with stage T1 and 41 patients with stage T2 tumors. The median interval between diagnosis and surgery was 21 days. There were no significant differences in age, sex, Eastern Cooperative Oncology Group (ECOG) performance status, body mass index, tumor size, surgical approach, surgical procedure, pathological subtype, tumor stage, tumor grade, and residual tumor among the three groups. Overall survival(OS) and cancer-specific survival (CSS) were comparable; the 5-year OS of the short-, intermediate-, and long-wait time groups were 84.2%, 82.0%, and 89.8%, respectively (P=0.732). The 5-year CSS rates of the short-, intermediate-, and long-wait time groups were 87.1%, 88.9%, and 90.4%, respectively (P=0.896). Multivariate analysis revealed that wait time was not an independent prognostic factor for OS or CSS.

**Conclusion:**

Prolonged surgical wait time (> 90 days) does not influence survival in patients with clinically localized renal cell carcinoma.

## Introduction

Many factors, including assessment and treatment of comorbidities before surgery, patients’ attitude, capacity of high-volume centers, can affect the interval between diagnosis and curative surgery in cancer patients. Prolonged wait time between initial diagnosis and surgical removal of the tumor is relatively common in high-volume centers due to the large number of patients who are referred to them. Nearly 70% of patients with sporadic renal cell carcinoma (RCC) are incidentally diagnosed at health check-ups. In this condition, preoperative wait time seems appropriate for patients without any clinical symptoms or who are diagnosed at early stages with relatively small tumor sizes (1).

Many patients are concerned that a long wait time before surgery can lead to tumor progression and poor survival. The relationship between a long surgical wait time and poor prognosis in different cancers is controversial (2–5). In patients with small RCC (<T1a) with slow tumor growth, the intervals between initial imaging diagnosis and surgical treatment have been reported to be over 2 years; however, cancer-specific survival (CSS) is not adversely affected (6). In 2006, experts from the Canadian Surgical Wait Times Initiative recommended a maximum preoperative wait time of 90 days for patients with T1a RCC (7). These conflicting reports suggest that the effect of prolonged wait time on RCC prognosis is still uncertain.

Therefore, we aimed to assess the influence of surgical wait time on the survival of patients with clinically localized RCC.

## Patients and Methods

### Definition of Surgical Wait Time

Surgical wait time was defined as the interval between initial imaging diagnosis of RCC and nephrectomy. Most RCCs are detected incidentally by non-invasive imaging. Due to the high diagnostic accuracy of abdominal imaging, preoperative renal tumor biopsy is not recommended for patients with localized RCC who are scheduled to undergo surgery, especially in patients with clear findings on imaging examinations (8).

Our department conducted about 200 surgeries (partial or radical nephrectomy) per year in recent years. All of the RCC patients are treated equally. Once the preoperative examination is completed and the surgical contraindications are eliminated, we will arrange the surgery soon. The patient’s surgical schedule will not be subject to the conflict of the operation day. In this study, patients were stratified into the following three groups based on surgical wait time: short-wait group (≤ 30 days), intermediate-wait group (> 30 and ≤ 90 days), and long-wait group (>90 days). Their clinicopathological characteristics were evaluated, and the survival rates of the three groups were compared.

### Patients and Data Collection

We retrospectively evaluated the medical records of 561 consecutive patients who underwent nephrectomy for clinically localized RCC (T1-2N0M0) between July 2011 and March 2017. We collected clinicopathologic data, including age at initial imaging diagnosis of RCC, sex, Eastern Cooperative Oncology Group (ECOG) performance status (ECOG PS), and body mass index (BMI). The study was approved by the institutional review board from The Affiliated Hospital of Xuzhou Medical University (A tertiary hospital with more than 4000 beds). Written informed consent was obtained from the patients for the publication of this study.

In order to confirm the original diagnosis, we invited an experienced urological pathologist to check all pathological specimens again. The tumor stage was determined based on the American Joint Committee on Cancer TNM staging system for kidney cancer (8th, 2017). Tumor grade was defined based on the 2016 World Health Organization classification grading system.

### Statistical Analysis

Kruskal-Wallis test and one-way ANOVA test were conducted to compare continuous variables. Kaplan-Meier survival curve analysis was performed to estimate overall survival (OS) and CSS, and a log-rank test was applied to compare differences among the three groups. OS was calculated from the date of surgery to date of last contact or date of death. CSS was defined as death primarily due to metastatic RCC. Univariate and multivariate Cox proportional hazards models were used to identify independent prognostic factors for OS and CSS. A two-sided p-value of <0.05 was considered statistically significant. All statistical analyses were conducted using SPSS version 20.0 (IBM Corporation, Armonk, NY, USA).

## Results

**Figure 1:**
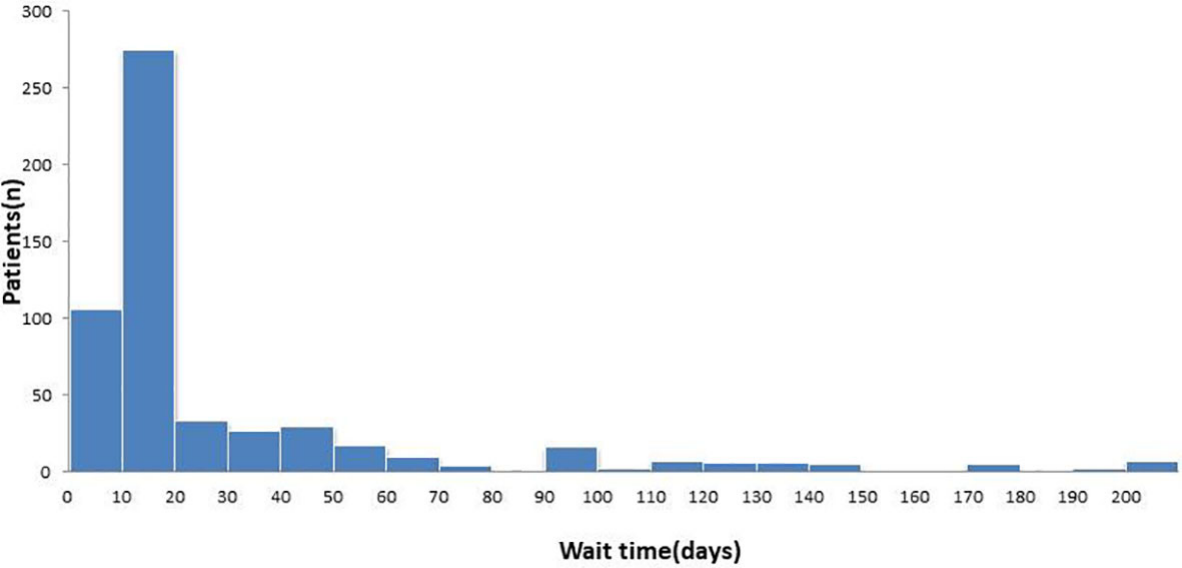
Wait time from initial diagnosis to surgery.

A total of 561 RCC patients were included in the present study. The intervals between initial imaging diagnosis of RCC and nephrectomy are shown in **Figure 1**. The median preoperative wait time was 21 days. The clinical features of the patients are shown in **Table 1**. Of the 561 patients, 416 were classified into the short-wait group (74.2%), 85 into the intermediate-wait group (15.2%), and 60 into the long-wait group (10.7%). The median surgical wait times of the short-, intermediate-, and long-wait groups were 14, 50 and 130 days, respectively. There were no significant differences in age, sex, ECOG PS, and BMI among the three groups. **Table 2** summarizes the surgical and pathological results. The numbers of patients with stage T1 and T2 were 520 and 41, respectively. The median tumor sizes of the short-, intermediate-, and long-wait groups were 4.2cm, 4.7cm and 4.5cm, respectively. Overall, 158 patients (28.2%) experienced tumor growth between imaging and nephrectomy (45, 62, and 51 patients in the short-, intermediate-, and long-wait groups, respectively). One patient in the long-wait group presented with an advance in pathological stage.

**Table 1:** Clinical features of patients in the short-, intermediate-, and long-wait groups.

	Short (n = 416)	Intermediate (n = 85)	Long (n = 60)	P
Age(yr)	62(20-85)	64(26-79)	67(27-73)	0.175
Gender				0.707
Male	275	58	37	
Female	141	27	23	
ECOG performance status				0.715
0	402	83	59	
1	14	2	1	
Body mass index(kg/m^2^)				0.120
Median	22.5(20.4-24.7)	22.8(19.7-25.3)	22.4(20.1-24.9)	
Median wait time(d)	14	50	130	

ECOG, Eastern Cooperative Oncology Group.

**Table 2:** Comparison of surgical and pathological results of patients in three groups.

	Short(n = 416)	Intermediate(n = 85)	Long(n = 60)	P
Size(cm)	4.2 ± 2.1	4.7 ± 2.4	4.5 ± 1.4	0.058
Absolute tumor growth (N=178),cm, median	0.1	0.2	0.5	0.01
Surgical approach				0.721
Open	47	8	5	
Laparoscopy	369	77	55	
Surgical procedure				0.846
RN	250	53	38	
PN	166	32	22	
Histological type				0.266
ccRCC	379	71	53	
pRCC	15	8	5	
chRCC	20	5	2	
mcRCC	2	1	0	
Grade				0.112
1	62	10	5	
2	330	64	53	
3	21	10	1	
4	3	1	1	
T stage				0.124
T1	385	76	59	
T2	31	9	1	
Residual tumor				0.889
R0	159	30	21	
R1	7	2	1	

RN, Radical nephrectomy; PN, Partial nephrectomy; ccRCC, Clear cell renal cell carcinoma; pRCC, Papillary renal cell carcinoma; chRCC, Chromophobe renal cell carcinoma;

mcRCC, Multilocular cystic renal neoplasm of low malignant potential.

The choice of surgical procedure was based on tumor size, degree of exophytic status, and proximity of the tumor to the collecting system or renal sinus. In the short-wait group, 250 patients (60.1%) underwent radical nephrectomy (RN) and 166 (39.9%) underwent partial nephrectomy (PN). In the intermediate-wait group, 53 patients (62.4%) underwent RN and 32 (37.6%) underwent PN. In the long-wait group, 38 patients (63.3%) underwent RN and 22 (36.7%) underwent PN. There were no significant differences in surgical approach and procedure, histological subtype, pathological grade, T stage, or residual tumor among the three groups. R0 resection was achieved in most patients who underwent PN. However, R1 resection was performed in ten patients (4.5%), and all of them were managed with close follow-up rather than additional RN.

**Figure 2:**
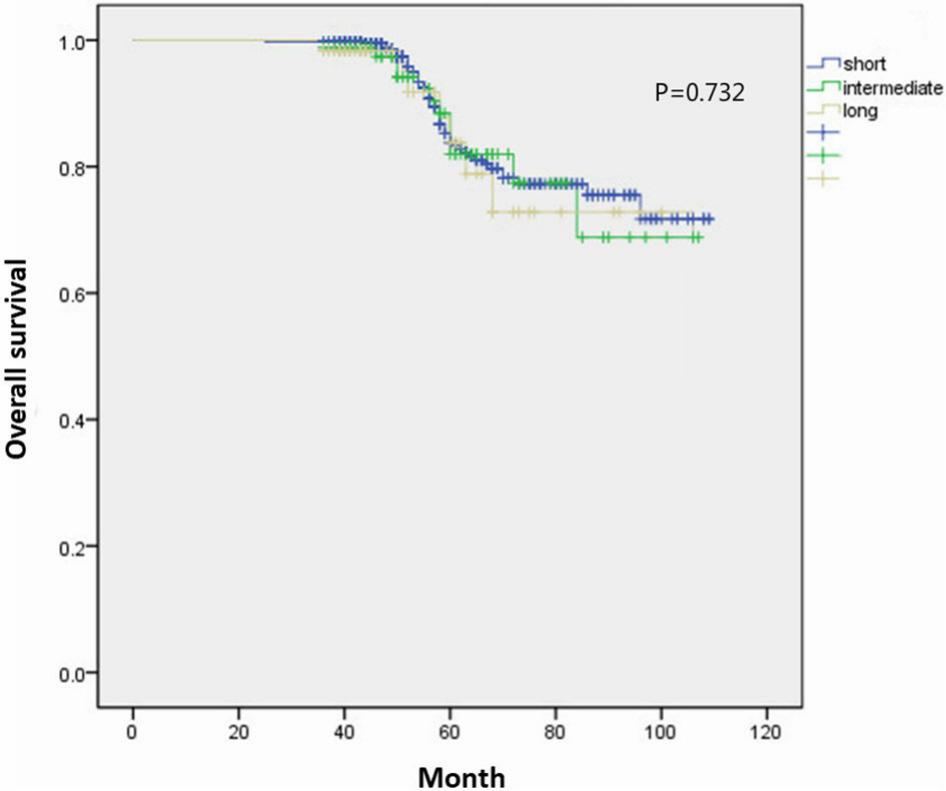
Comparison of overall survival curves between patients with different wait groups.

All patients were closely followed for a median time of 56 months (range, 25-109 months). Disease recurrence rates in the short-, intermediate-, and long-wait time groups at 5 years after surgery were 21%, 24%, and 23%, respectively. The 5-year OS of all patients was 83.8%. The 5-year OS rates of the short-, intermediate-, and long-wait time groups were 84.2%, 82.0%, and 89.8%, respectively (P=0.732) (**Figure 2**). The 5-year CSS rates of the short-, intermediate-, and long-wait time groups were 87.1%, 88.9%, and 90.4%, respectively (P=0.896). After adjusting for sex, ECOG PS, wait time, histological type, and pathological grade, we found no evidence that surgical wait time was associated with decreased CSS or OS (**Table 3**).

**Table 3:** Multivariable Cox model for cancer-specific survival and overall survival.

	Cancer-specific survival			Overall survival		
	HR	95% CI	*p* value	HR	95% CI	*p* value
Gender			0.286			0.365
Male	Ref.			Ref.		
Female	1.108	0.782-2.431		1.251	0.771-2.028	
ECOG PS			0.503			0.467
0	Ref.			Ref.		
1	2.162	0.725-4.882		1.548	0.477-5.026	
Wait group			0.582			0.453
Short	Ref.			Ref.		
Intermediate	1.163	0.642-1.893		1.098	0.581-2.076	
Long	1.418	0.832-2.573		1.628	0.761-3.483	
Histological subtype			0.781			0.959
ccRCC	Ref.			Ref.		
Non-ccRCC	1.33	0.431-3.256		0.988	0.623-1.568	
Grade			0.105			0.486
G1	Ref.			Ref.		
G2	1.781	0.643-2.671		1.177	0.745-1.859	
G3/4	2.162	0.853-4.192		1.823	0.648-3.304	
T Stage			0.003			<0.001
T1	Ref.			Ref.		
T2	2.331	1.447-4.074		3.399	1.972-5.859	

## Discussion

Surgery is the only curative treatment for localized RCC. Once detected by imaging examination, RCC is primarily treated by surgery. However, prolonged wait time between initial diagnosis and surgical removal of the tumor is a common phenomenon, especially in high-volume centers. The healthcare systems of different areas are organized in different ways. Due to the large number of patients referred to these centers and the limited hospital bed capacity, a certain wait time before admission to high-volume centers is usually mandatory (9, 10). In addition to social factors, the self-factor of cancer patients may also affect preoperative wait time. Without any symptoms or discomfort, the patient in the early stage may not take imaging results seriously and lack willingness or knowledge to seek further medical attention. Furthermore, comorbidities, such as acute cerebral/myocardial infarction, may be contraindications to surgery. Older patients or those with more comorbidity were significantly more likely to undergo initial treatment > 30 days from diagnosis. Patients with early stage cancer had longer wait times compared to that of patients with advanced-stage cancer (11).

Surgical wait time can also be affected by the surgical approach. The role of robot-assisted surgery in the management of RCC has exponentially grown in the past 10 years. In patients with RCC, robot-assisted RN shows perioperative advantages compared to open RN. Compared to laparoscopic RN, robot-assisted RN provides similar surgical outcomes (12). The long-term oncologic and functional outcomes of robot-assisted PN are excellent (13). However, if all surgeries were performed robotically, an additional 32 days would be needed, which could significantly increase the surgical wait time (14).

Some previous reports have found that prolonged wait time and delay in surgery lead to cancer progression. Waldert et al. reviewed the records of 187 upper-tract urothelial carcinoma patients who underwent radical nephroureterectomy (RNU). They found that a delay from diagnosis to RNU, analyzed as a continuous variable, was associated with advanced stage and higher grade. A surgical wait time of more than 3 months could lead to tumor upstaging (15). For RCC, the rate of tumor growth is related to the primary size of the tumor and is not affected by other factors including histological subtype, tumor grade, or patient age. A larger tumor at initial diagnosis will grow faster than a smaller tumor. It has been reported that the tumor sizes increased by an average of 0.13 cm annually for small renal masses (≤4 cm). Some RCCs even decrease in size over time. The likelihood of cancer progression to higher stages is low, and metastases are extremely rare (16). RCCs of <7 cm have a median tumor growth of only 0.4 cm annually (6). As the tumor growth rate of localized RCC is relatively slow, an appropriate delay in surgical wait time rarely leads to cancer progression.

There are no consistent results regarding the relationship between surgical wait time and the prognosis of different cancers. For patients with colorectal or breast cancers, a surgical delay of more than 12 weeks for curative surgery after initial diagnosis is associated with a poor prognosis. However, no obvious pattern of increased risk of all-cause mortality was observed in lung or thyroid cancers (17). Gore et al. identified 441 patients diagnosed with muscle-invasive bladder cancer in the Surveillance, Epidemiology, and End Results -Medicare dataset. Their results confirm that it is important to expedite treatment for a patient scheduled to undergo radical cystectomy, since a delay of >12 weeks increases the risk of cancer-specific and all-cause mortality in these patients (18). Korets et al. evaluated the effect of delaying surgery on radical prostatectomy outcomes. They retrospectively reviewed 1568 patients who underwent radical prostatectomy. Clinical and pathological results were compared between patients with ≤ 60, 61-90, and >90 days interval between surgery and time of prostate biopsy. They found that a delay of >60 days was not associated with adverse pathological outcomes in men with localized prostate cancer, nor did it correlate with poor biochemical recurrence-free survival (19). Therefore, there are different results with respect to the relationship between prolonged surgical wait time and adverse prognosis for cancer. This may be attributed to the different intrinsic features of and surgical approaches for different cancers.

The present study found that a wait time of more than 3 months from imaging diagnosis to nephrectomy for localized RCC was not associated with decreased survival. Similarly, a recent study shows that surgical wait times up to 24 weeks are not associated with adverse oncologic outcomes for RCC (20). However, our findings do not suggest that curative surgery should be delayed for prolonged periods of time without good reasons. In any case, the surgery of a cancer patient is a confined operation. The period between initial diagnosis and curative surgery may be difficult for cancer patients. Some researchers found that cancer patients experienced an increased risk of multiple mental disorders from the time of initial cancer diagnosis (21, 22). Several studies have evaluated the influence of the interval between initial diagnosis and final treatment on the psychological health of cancer patients. For patients with newly diagnosed esophageal or gastric cancer, longer wait time was associated with more frequent psychiatric hospital care during the first year after treatment among patients with preexisting mental disorders (23). Lally proposed that a certain time between cancer diagnosis and treatment initiation might be necessary and allow psychological adjustment for breast cancer patients (24). In summary, if a certain time period is required to optimize comorbidities before curative surgery for cancer patients, we should monitor their mental state and consider surgery immediately after controlling these comorbidities.

There are some limitations in our study. The numbers of patients in the intermediate- and long-wait groups were relatively small. Owing to its retrospective nature, the study lacked randomization, and selection bias is inevitable. Many factors may affect the preoperative wait time from the initial imaging diagnosis. In addition, the distributions of wait times were relatively varied in the long-wait group. Further study is required to confirm the safety of prolonged surgical wait time for clinically localized RCC.

## Conclusion

In patients with clinically localized RCC, a wait time of more than 3 months from diagnosis to nephrectomy may not adversely influence OS or CSS. A certain delay in order to optimize comorbidities, or to improve physical condition, is acceptable.

## Data Availability Statement

The raw data supporting the conclusions of this article will be made available by the authors, without undue reservation.

## Ethics Statement

The studies involving human participants were reviewed and approved by Affiliated Hospital of Xuzhou Medical University. The patients/participants provided their written informed consent to participate in this study.

## Author Contributions

NQ, FZ, and XL conceived the study, participated in its design, collected the data, performed the statistical analysis, and drafted the manuscript. WW and JW participated in its design, and helped to draft the manuscript. All authors contributed to the article and approved the submitted version.

## Funding

This work was supported by the 2018 Doctoral Project for Innovation and Entrepreneurship of Jiangsu Province and the Natural Science Foundation Youth Project of Jiangsu Province (BK20190989).

## Conflict of Interest

The authors declare that the research was conducted in the absence of any commercial or financial relationships that could be construed as a potential conflict of interest.
